# *QuickStats:* Death Rates* for Motor Vehicle Traffic Injury,^†^ by Age Group — National Vital Statistics System, United States, 2015 and 2017

**DOI:** 10.15585/mmwr.mm6806a8

**Published:** 2019-02-15

**Figure:**
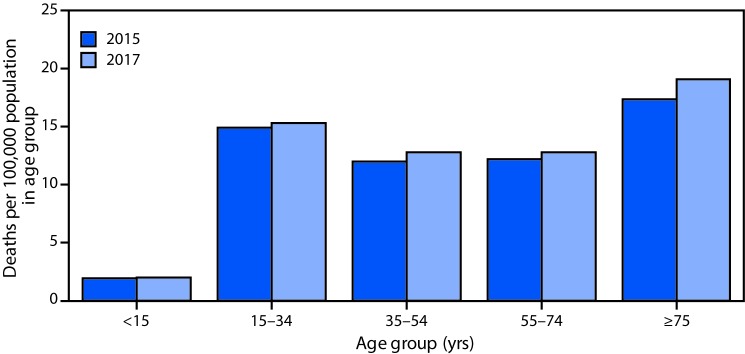
From 2015 to 2017, death rates for motor vehicle traffic injury increased for persons aged ≥15 years. For infants and children aged <15 years there was no statistically significant change from 2015 to 2017, and this group had the lowest death rate (2.0 deaths per 100,000) in 2017. The highest death rate in 2017 was for persons aged ≥75 years (19.1), followed by a 15.3 death rate for persons aged 15–34 years, and 12.8 for persons aged 35–54 and 55–74 years.

**Source:** National Vital Statistics System. Underlying cause of death data, 1999–2017. https://wonder.cdc.gov/ucd-icd10.html.

For more information on this topic, CDC recommends: https://www.cdc.gov/motorvehiclesafety/index.html.

